# A Multimodal Messaging App (MAAN) for Adults With Autism Spectrum Disorder: Mixed Methods Evaluation Study

**DOI:** 10.2196/33123

**Published:** 2021-12-07

**Authors:** Mohamad Hassan Fadi Hijab, Dena Al-Thani, Bilikis Banire

**Affiliations:** 1 Division of Information and Computer Technology College of Science and Engineering Hamad Bin Khalifa University Doha Qatar

**Keywords:** autism, assistive technology, mobile app, social and communication skills

## Abstract

**Background:**

Individuals with autism spectrum disorder (ASD) often exhibit difficulties in social and communication skills. For more than 30 years, specialists, parents, and caregivers have used techniques, such as applied behavioral analysis, augmentative and alternative communication, and the picture exchange communication system to support the social and communication skills of people with ASD. Even though there are many techniques devised to enhance communication, these techniques are not considered in existing social media apps for people with ASD.

**Objective:**

This study aimed to investigate the effect of adding accessibility features, such as text-to-speech (TTS), speech-to-text (STT), and communication symbols (CS), to a messaging app (MAAN). We hypothesized that these accessibility features can enhance the social and communication skills of adults with ASD. We also hypothesized that usage of this app can reduce social loneliness in adults with ASD.

**Methods:**

Semistructured interviews were conducted with 5 experts working in fields related to ASD to help design the app. Seven adults with ASD participated in the study for a period of 10 to 16 weeks. Data logs of participants’ interactions with the app were collected. Additionally, 6 participants’ parents and 1 caregiver were asked to complete a short version of the Social and Emotional Loneliness Scale for Adults (SELSA-S) questionnaire to compare pre-post study results. The Mobile Application Rating Scale: user version questionnaire was also used to evaluate the app’s usability. Following the study, interviews were conducted with participants to discuss their experiences with the app.

**Results:**

The SELSA-S questionnaire results showed no change in the family subscale; however, the social loneliness subscale showed a difference between prestudy and poststudy. The Wilcoxon signed-rank test indicated that poststudy SELSA-S results were statistically significantly higher than prestudy results (*z*=−2.047; *P*=.04). Point-biserial correlation indicated that the SELSA-S rate of change was strongly related to usage of the TTS feature (*r*=0.708; *P*=.04) and CS feature (*r*=−0.917; *P*=.002), and moderately related to usage of the STT feature (*r*=0.428; *P*=.17). Lastly, we adopted grounded theory to analyze the interview data, and the following 5 categories emerged: app support, feature relevance, user interface design, overall feedback, and recommendations.

**Conclusions:**

This study discusses the potential for improving the communication skills of adults with ASD through special features in mobile messaging apps. The developed app aims to support the inclusion and independent life of adults with ASD. The study results showed the importance of using TTS, STT, and CS features to enhance social and communication skills, as well as reduce social loneliness in adults with ASD.

## Introduction

Autism spectrum disorder (ASD) is a neurodevelopmental disorder that is characterized by repetitive patterns of behavior, restricted interest, and deficits in social and communication skills [1]. These deficits affect the mental development of people with ASD, especially their socioemotional development, which may result in social and family loneliness [2]. Loneliness can be described as a lack of a close or intimate attachment to family members and friends. The consequences of loneliness include feelings of emptiness, anxiety, depression, suicide, and anger, which are detrimental to both physical and mental health [3]. Another effect of loneliness is learning disabilities, as the deficits associated with ASD limit patients’ opportunities to interact with their social environment and result in delays in learning social norms [3]. Understanding social norms is essential for positive and long-lasting social relationships. For example, an individual with ASD might not know when someone is angry and how to reciprocate. This lack of social norms may lead to social rejection. Social and communication skills are thus crucial components for social and emotional development. In recent years, the ASD prevalence rate has increased, with significant variability worldwide. In the United States, 185 in 10,000 children have a diagnosis of ASD [4]. Similarly, recent prevalence studies in the Middle East reported that 114 in 10,000 children from Qatar [5], and between 49 and 513 in 10,000 children from Lebanon have the disease [6]. Given the increasing prevalence of ASD, the impact of problems in social and communication skills associated with this disorder may lead to a high societal cost of supporting the physical and mental development of individuals with ASD. Thus, it is critical to develop innovative communication platforms to enhance social and communication skills between individuals with ASD and others.

Many interventions have been used to enhance social and communication skills, such as applied behavioral analysis (ABA) [7], augmentative and alternative communication (AAC) [8], and the picture exchange communication system (PECS) [9]. ABA is known as the primary treatment for children with ASD [10]. It is considered necessary for education and behavioral interventions, as it is commonly used to assist and improve social and communication skills for children with ASD. AAC provides individuals with ASD with pictures and illustrations to express their needs, and it is extensively used, although some caregivers and specialists have expressed concerns regarding its application. They often highlight that usage of AAC will prohibit the development of verbal speech in individuals with ASD [11]. Nevertheless, these technologies have the potential for supporting the social and communication skills of individuals with ASD [12]. The number of studies using AAC has increased due to the rise in its demand and value for an assistive technology tool in the last few years [13]. Lastly, the PECS is considered as one of the forms of AAC to help individuals with ASD to communicate. These systems appear promising due to their ability to construct sentences using images and symbols [14], with a low requirement of minor motor planning skills and little cognitive demand, which is why limited training is needed [15]. Many studies have investigated the importance of the PECS and determined its major role in enhancing social and communication skills [14,16]. On the other hand, PECS has several disadvantages, including difficulties in virtual communication and low speed in constructing messages [15]. However, behavioral and educational interventions, and digital revolutions have tremendously influenced the lives of individuals with ASD positively [17]. These technologies have increased the utilization of virtual communication platforms and highlighted the importance of providing remote services to the population with ASD [18]. Moreover, technology can ease communication and enhance individuals’ social well-being [19]. The use of assistive technologies and tools can help create a platform that allows individuals with ASD to share their thoughts, ideas, and emotions effortlessly [19]. These technologies were introduced to support their communication challenges, and help them interact and express their feelings with the world without physical presence [20].

This study intends to verify the effectiveness of a new mobile messaging app called “MAAN,” which means “together” in Arabic, for adults with ASD, using pre-post study data. The data were collected using questionnaires, interviews, and interaction logs. The aim of MAAN is to provide an accessible, safe, and easy-to-use messaging app for adults with ASD and hence support their inclusion in society. In this study, we hypothesized the following: (1) The accessibility features of mobile apps (text-to-speech [TTS], speech-to-text [STT], and communication symbols [CS]), which are applied to MAAN, can enhance the social and communication skills of adults with ASD; and (2) The usage of the app can reduce social loneliness in adults with ASD.

## Methods

### Study Design

A mixed methods approach was followed in this study. Seven adults with ASD were asked to use MAAN with their caregivers for 16 weeks. Prior to the design and development of MAAN, semistructured interviews were conducted with experts to discuss the preliminary proposed app design. The app design was then modified according to the received feedback. After that, adults with ASD and their parents or caregivers were asked to use MAAN for a period of 16 weeks. This duration was adopted from the study by Laugeson et al [21], which suggested that when receiving social and communication skills interventions, improvement is only evident at 16 weeks. The study started by contacting individuals willing to participate. Due to COVID-19, the initial meetings with the participants were conducted online. All participants received an email for explaining the study information, such as date and time, and a weblink for collecting informed consent for a virtual meeting with the participants, their friends, and their parents. After receiving the participants’ consent, they were asked to fill an online prestudy questionnaire in order to collect demographical information. The parents or caregivers of the participants were also asked to complete a short version of the Social and Emotional Loneliness Scale for Adults (SELSA-S) questionnaire [21]. The aim of this questionnaire was to determine the social and family loneliness of the participants. In a virtual meeting, the principal researcher explained the app features in detail and answered further questions regarding the study. The participants were also provided with a hotline to report any issue they might face during this study. To ensure the continuity of data collection, the participants were asked to make sure that the app was constantly running in the background.

At the end of the study, all participants with ASD and their caregivers or parents completed the Mobile Application Rating Scale: user version (uMARS) [22] questionnaire. The uMARS is a simple and reliable tool that can be used by end-users to assess the quality of mobile health apps. It provides a 20-item measure that includes 4 objective quality subscales (engagement, functionality, esthetics, and information quality) and 1 subjective quality subscale. The purpose of this questionnaire is to measure the app’s quality in terms of usability. The participants’ parents or caregivers also completed the SELSA-S questionnaire again to determine the participants’ social and family loneliness rates after the study. Lastly, a semistructured interview with parents or caregivers was conducted to gather information about the context of app usage. The interview included questions about the overall app experience in terms of usability, functionality, and challenges encountered while using the app, as well as suggestions for further enhancement.

### MAAN App Design and Development

The app consisted of the following 3 main features: TTS, STT, and CS. One of the well-known features that can also be considered as a type of assistive technology is the TTS feature, which allows users to listen to written text on a computer, tablet, or smartphone. This technology is popular among people having difficulties in reading and decoding words [23]. Similarly, STT is an assistive technology feature used by people who struggle with writing. This form of assistive technology allows speech to be converted into text using the computing device. Both features are very commonly employed to support inclusion education settings [24]. The app also included the CS feature, which is an essential part of AAC-supported technologies, and its primary purpose is to assist individuals with ASD in constructing sentences. We are not aware of any messaging app that supports the use of the CS feature. “TAWASOL symbols” were employed for the CS feature. They were developed in 2013 by Mada Center, an assistive technology center in Qatar. These symbols represent the Modern Standard Arabic language and are designed to be culturally, socially, religiously, and linguistically acceptable. TAWASOL is based on the AAC Symbols Collection and is directed to nonverbal or minimally verbal individuals who require alternative communication solutions [25].

**Figure 1:**
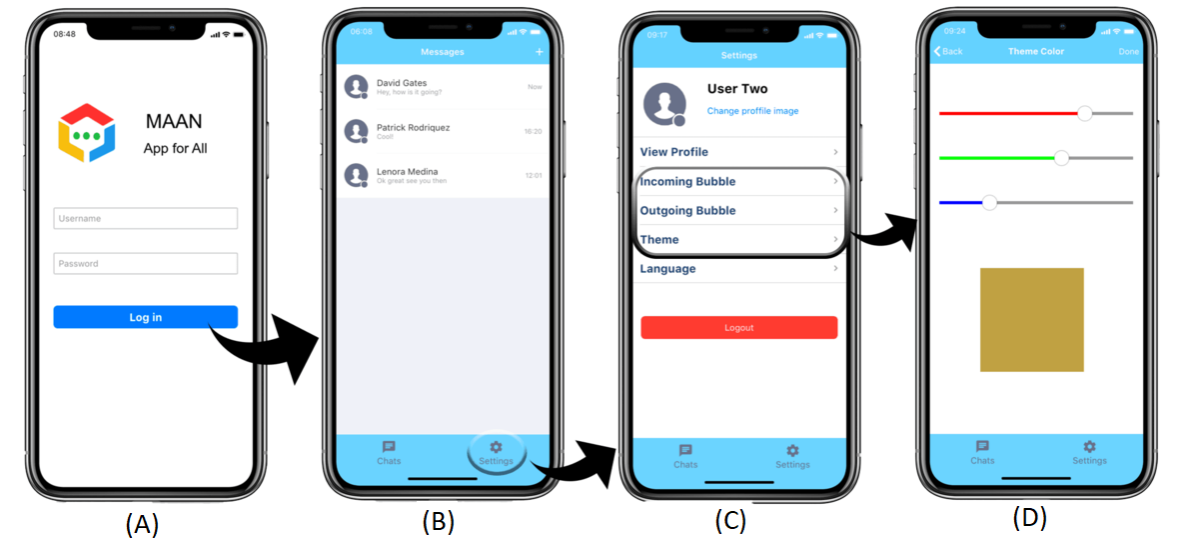
Screenshots of the MAAN app’s administrative section.

**Figure 2:**
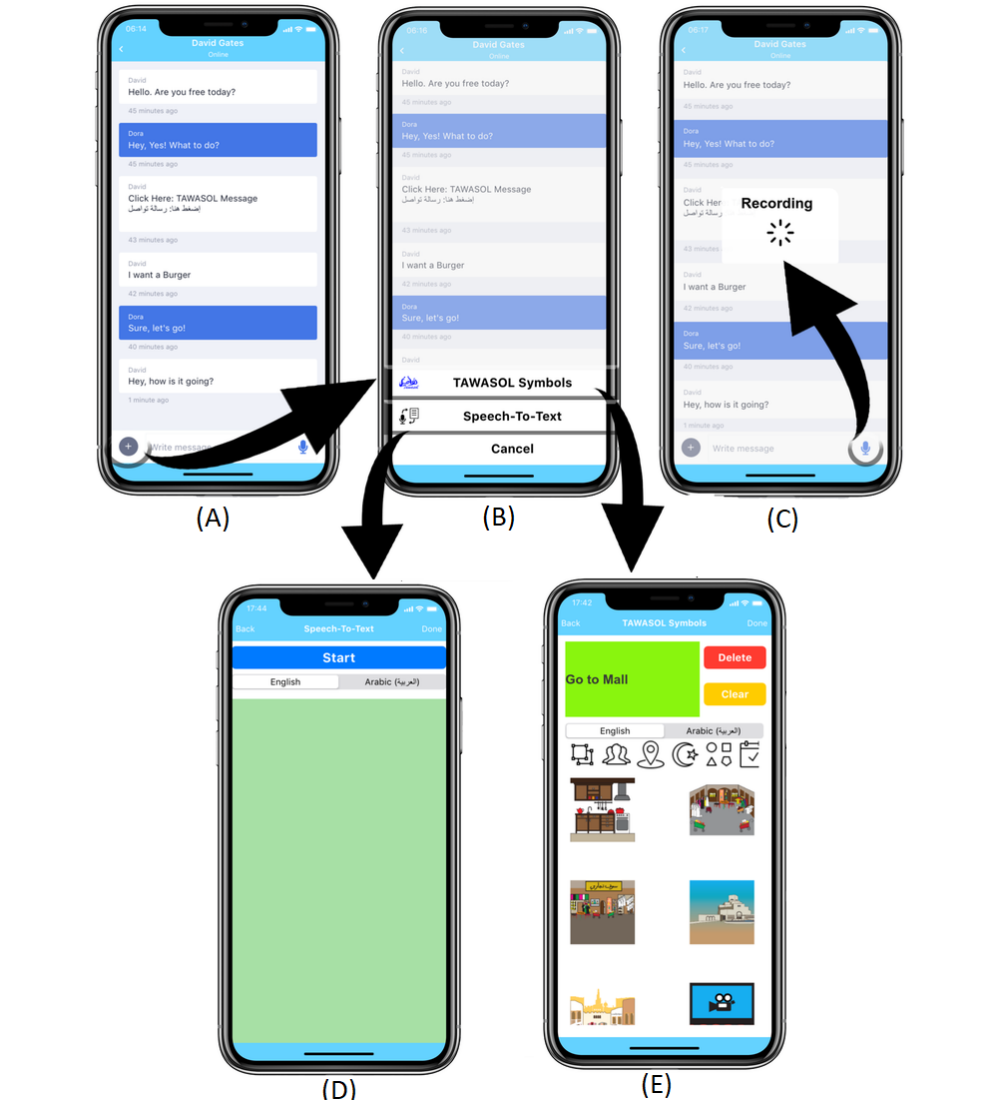
Screenshots of the MAAN app’s feature display screens.

MAAN was developed using Xcode, which is an Apple integrated development environment (IDE) for developing iOS-based software. MAAN is available on iOS devices and is designed with a minimal number of screens. The screen designs are similar to traditional and commonly used messaging apps. Thus, in order to provide individuals with a consistent and effortless experience, TTS and STT features were implemented in the app using Xcode libraries. Additionally, 478 images from TAWASOL symbols were divided into 25 categories and added to MAAN. The essence of the category was to reduce the search time of required symbols. Participants can click on a certain category and then choose a TAWASOL symbol. The textual description of the selected TAWASOL symbol will be displayed in both Arabic and English. The user can specify the language of the symbol textual description, which is another advantage of MAAN over other apps. When previewing the received symbol, the user can listen to the TAWASOL symbol textual description. A challenging aspect related to designing for adults with ASD is the choice of the interface color theme [26,27]. MAAN aims to address this issue by allowing users to choose their suitable color theme. The MAAN app flow can be divided into the following 2 sections: administrative and features. Figure 1 shows screenshots of the app’s administrative flow, and Figure 2 shows screenshots of the app’s features.

In the administrative section, the app starts with a login screen where the participants can login using an assigned username and password, and remain logged in until they logout manually (Figure 1A). The participants can select whom to chat with and start their conversation (Figure 1B). The setting screen provides the participants with the ability to view their profile, and change the app theme color and the incoming and outgoing messaging bubble color (Figure 1C and Figure 1D). Moreover, the participants can change the language of the app (Arabic or English) and logout from the app (Figure 1C).

The participants can use the features of the app as described in Figure 2. An accessory button in the chat window provides the participants with the choice to select the STT or TAWASOL symbols screen (Figure 2A and Figure 2B). On clicking the mic icon, they can record their voice and send it as a voice note to the person they are chatting with (Figure 2C). On selecting STT, the participants proceed to another screen where they can chat, and the chat will be directly transcribed into text to be sent (Figure 2D). On selecting TAWASOL symbols, the participants can choose symbols from the 25 different categories, which will be generated as text to be sent (Figure 2E). The last feature, TTS, will be activated when the participants press on any text message. With this feature, the system will transcribe the text into voice and read it.

### Testing and Analysis Tools

Ethical approval was obtained from the Research Board of Qatar Biomedical Research Institute. Participation was entirely voluntary, and each participant was sent an approved informed consent form that contained all study details. The following 4 types of consent forms were sent based on participant category: (1) participant without ASD (including caregiver or parent) consent form, (2) specialist consent form, (3) verbal participant with ASD consent form, and (4) nonverbal participant with ASD consent form. Each participant was aware of the requirement to provide an informed decision regarding participation in the study, as well as the right to withdraw from the study without justification or penalties. Moreover, participants were assured about the confidentiality and security of the data collected. The user evaluation study was conducted in a real-life setting, and the participants were recruited through snowballing techniques [28]. As of November 2020, a total of 7 adults with ASD, aged between 18 and 30 years, were recruited from Qatar and Lebanon. For each participant, parents, caregivers, or specialists were recruited for using the app.

Quantitative and qualitative measures were collected in this research. In terms of quantitative measures, this study employed 3 questionnaires and collected the interaction log data through the app. The prestudy questionnaire was used to collect the participants’ demographic data, and the SELSA-S [29,30] questionnaire was used to determine social and family loneliness for participants prior to the study and after completing the study. The uMARS [31] questionnaire was used to measure the app’s quality after completing the study. Interaction log data were also collected through Google Firebase. This included the number of times the app was accessed, and how each feature was used.

Quantitative data were complemented with qualitative data collected through the poststudy interview. The aim of the interview was to produce contextual real-world knowledge about the behaviors and social structures of the participants, and their experiences with the MAAN app in their daily lives. The interview was conducted with parents or specialists of the participants and was regarding social and communication skills development.

#### Quantitative Analysis

The first questionnaire used was the SELSA-S questionnaire. The full version of the SELSA questionnaire is a 37-item self-report measure of romantic, social, and family loneliness. It was administered to young adults with ASD prestudy and poststudy [29,30]. Moreover, the Program for Education and Enrichment of Relational Skills mainly used it in most of their studies [21] to track the social and loneliness aspects of their participants. A study showed that the SELSA-S questionnaire, which consists of 15 items, is a psychometrically reliable and valid alternative to the full version of SELSA, and requires less time for participants [32]. There are 5 items referring to the family loneliness subscale, 6 items referring to the romantic subscale, and 4 items referring to social loneliness [33]. All items are answered on a 7-point Likert scale. This study used only the social and family subscales, since emotional change was not in the interest of this study. The SELSA-S questionnaire was used prestudy and poststudy, and the results were compared to find the rate of change.

The Wilcoxon signed-rank test is a nonparametric test that is particularly suitable for examining the difference between pretest and posttest measures in a small sample [34], and it was used in this study. Statistical analysis was performed using IBM SPSS software (IBM Corp), and a *P* value <.05 was considered statistically significant.

The second questionnaire was the uMARS questionnaire that was coded following the scoring guides provided by the questionnaire developers [22]. The questionnaire consists of the following 3 sections: app quality, app subjective quality, and perceived impact. Moreover, the rating is scored out of 5, based on the scoring criteria given by the questionnaire developers. This questionnaire was used poststudy to evaluate the app’s quality. Descriptive statistical analysis of the uMARS rating was used to gain insights into the usability experience of the participants with ASD. Many studies have used the uMARS questionnaire to evaluate the usability of developed apps [35-37].

#### Qualitative Analysis

To analyze the interviews, open and axial coding phases from the grounded theory were used. Grounded theory is a systematic methodology extensively used in qualitative research. This approach aims to generate a substantive theory that links the investigated data to reality [38]. After conducting the poststudy interview with the participants, the interview was transcribed by the first author (MHFH). Open coding was then used to generate initial concepts from the data. This was followed by axial coding to establish connections between different concepts and categories [39].

Prestudy and poststudy interviews were conducted. The prestudy interview was conducted with 5 experts, 2 of whom worked in special educational programs (specialist and educational and ABA/applied verbal behavior consultant, and Information and Communications Technology access expert in educational programs). The other 3 worked in directing centers for adults with ASD (director of the Rehabilitation and Inclusion Office, manager of the Severe Difficulties Department, and president of the Disability Association). The interviews were conducted for the purpose of obtaining insights into the current situation and existing technologies (state-of-the-art), and obtaining feedback on the study design. The poststudy interview was conducted with all participants’ parents or caregivers to gather information about the context of use. The interview included questions about the overall app experience in terms of usability, functionality, and challenges encountered while using the app, as well as suggestions for further enhancement.

## Results

### Demographic Information

Among the 7 adults with ASD, 5 contacted their parents through the app, 1 contacted both a parent and specialist (ABA specialist and teacher with 25 years of experience), and 1 contacted only a specialist (speech and language therapist with 5 years of experience) (Table 1). Moreover, there was diversity in educational background among the adults with ASD. This diversity in educational background was related to the severity of their conditions. Most of the parents and caregivers interacted with the participants for less than 6 hours per day. Based on the information gathered from the participants’ parents, 2 participants were diagnosed with high-functioning ASD (participants 3 and 4), 3 were diagnosed with medium-functioning ASD (participants 5, 6, and 7), and 2 were diagnosed with low-functioning ASD and up to severe ASD (participants 1 and 2). Participant information is presented in Table 2.

**Table 1:** Demographic information.

Characteristic			Adults with autism spectrum disorder (N=7), n		Parents (N=6), n		Specialists (N=2), n
**Gender**							
	Female	0		5		2	
	Male	7		1		0	
**Age (years)**							
	18-21	4		0		0	
	21-26	1		1		0	
	>26	2		5		2	

**Table 2:** Characteristics of the participants with autism spectrum disorder.

Characteristic described by a parent or specialist	Participant
Completely nonverbal: Cannot speak but understands spoken conversation	Participants 1, 2, 3, and 5
Verbal: Can greet	Participant 4
Nonverbal: Cannot speak a lot and only greets, but understands spoken conversation	Participants 6 and 7

### Participant Engagement

The user study evaluation lasted 16 weeks. However, not all participants completed this duration due to this project’s time limitation. Three participants completed the 16-week duration, whereas 2 participants used the app for 13 weeks, 1 participant used it for 11 weeks, and 1 participant used it for 10 weeks. The full duration of the study was tailored to each participant according to their enrolled duration. In this study, the duration was defined as the period from the start day after enrollment and training to the day when the poststudy interview was conducted. Participants’ daily activities were monitored through interaction log data. Table 3 represents a summary of the average number of days the app was accessed by each participant per week. Although it was difficult to determine who initiated a conversation, since it could last or hold for a while, initiation was considered when the participant began the chat each day. Based on responses in the poststudy interview, 2 participants initiated some of the conversations, especially when waking up in the morning, needing something to eat, or needing to go out (requesting a need); 2 participants partially initiated conversations with some encouragement from their parents or caregivers; and 3 participants did not initiate any conversations.

**Table 3:** Average number of days per week the app was accessed by each participant.

Participant	Value (days/week)^a^, mean (SD)
1	5.00 (1.826)
2	2.37 (1.821)
3	2.87 (1.893)
4	4.62 (1.981)
5	1.69 (1.109)
6	3.36 (2.203)
7	2.10 (1.287)

^a^The overall maximum and minimum values were 5 and 1.69 days/week, respectively, and the overall mean value was 3.14 (SD 1.25) days/week.

**Figure 3:**
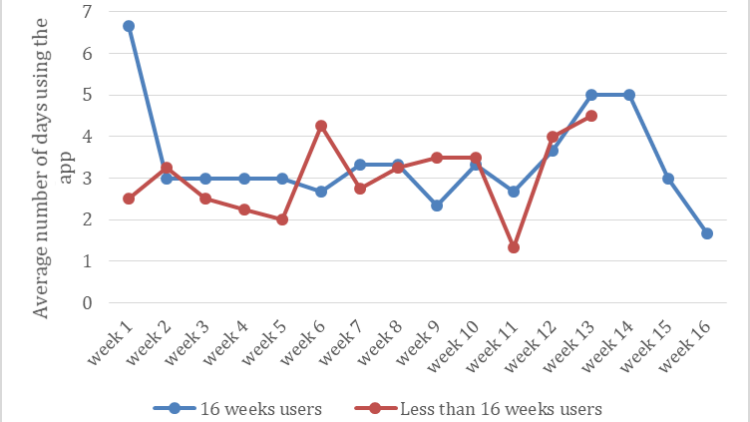
Participant engagement in the app.

Based on the collected data, diversity in participant engagement was noticed. The participants were categorized into the following 2 groups: participants who used the app for 16 weeks and participants who used the app for less than 16 weeks. Figure 3 shows the average participant engagement for the 2 groups.

### Testing and Analysis Tools

#### Quantitative Analysis

**Figure 4:**
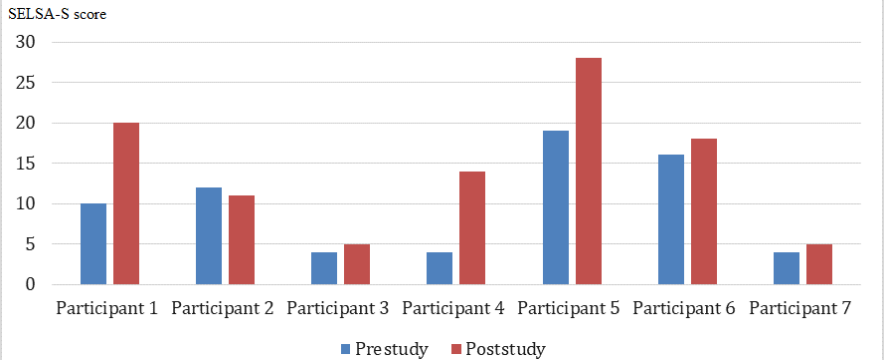
Prestudy and poststudy SELSA-S questionnaire social scale results. SELSA-S: short version of the Social and Emotional Loneliness Scale for Adults.

In this study, the social and family loneliness subscales were considered. It was evident from the collected data that the family loneliness subscale did not change from prestudy to poststudy. However, the social loneliness subscale showed a difference in all participants (Figure 4).

**Figure 5:**
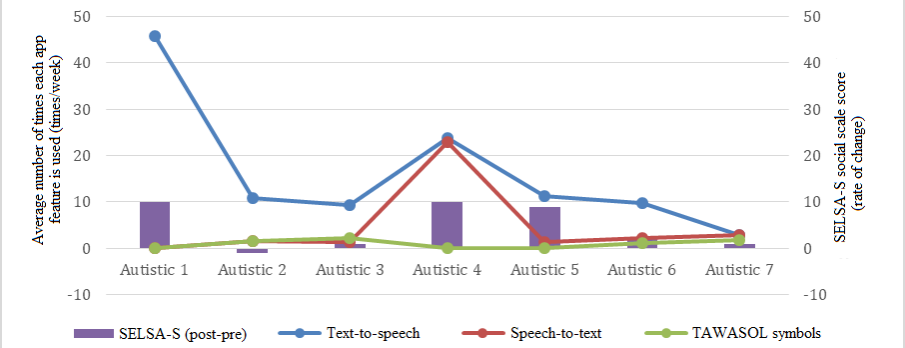
Average number of times each app feature is used per week with respect to the rate of change of the SELSA-S questionnaire result. SELSA-S: short version of the Social and Emotional Loneliness Scale for Adults.

The use of each app feature differed from one participant to another. However, it was evident that when the app features were frequently used, the SELSA-S rate of change tended to be higher (Figure 5). For example, participant 1 used TTS more often than all other participants, and thus, the SELSA-S rate of change of this participant was the highest. Participant 7 used the app features less frequently and did not get engaged with the app when compared with the other participants. The SELSA-S rate of change for this participant was low.

Regarding uMARS results, first, the app quality mean score was calculated by finding the mean of the ratings for the subsections engagement, functionality, esthetics, and information, and then calculating the average of the 4 means. The mean value was 3.62 (SD 0.65). Second, app subjective quality was rated based on the mean score of the related questions, which was 3.72 (SD 1.12). Third, the questionnaire’s perceived impact section consists of 6 questions that rate the impact of the app on the participants’ knowledge, attitude, and intention regarding the targeted health behavior. Moreover, the rating was based on the mean score of the related questions, which was 3.54 (SD 0.98).

Point-biserial correlation was employed to answer the first hypothesis. The number of times each app feature was used was considered a dichotomous variable, since it is either clicked or not [40,41]. Therefore, point-biserial correlation, which is a special case of Pearson correlation, was used to measure the strength of the association between the SELSA-S rate of change (post-pre) and each app feature. The strength of the correlation was assessed as follows: week correlation if 0.1<|r|<0.3, moderate correlation if 0.3<|r|<0.5 and strong correlation if 0.5<|r|. The results showed that the SELSA-S rate of change was strongly related to the TTS feature (*r*=0.708, *P*=.38) and to the TAWASOL symbols feature (*r*=−0.917, *P*=.002). On the other hand, the SELSA-S rate of change was moderately related to the STT feature (*r*=0.428, *P*=.17). Therefore, usage of TTS, STT, and CS (TAWASOL symbols) can enhance the social and communication skills of adults with ASD. For the second hypothesis, the Wilcoxon signed-rank test was used to determine whether the difference between the prestudy and poststudy results in the social loneliness scale was significant. It indicated that poststudy SELSA-S results were statistically significantly higher than prestudy SELSA-S results (*z*=−2.047; *P*=.04). Thus, the null hypothesis was rejected. Therefore, usage of the app can reduce social loneliness among adults with ASD.

#### Qualitative Analysis

All the interviewees in the prestudy interviews agreed on the importance of the MAAN app as a unique tool that could be of great use. They stated that this innovative app would assist centers, parents, and caregivers in staying connected with adults with ASD. They also believed that MAAN has the potential to enhance the social and communication skills of adults with ASD. Moreover, they asserted that such an app could support the inclusion of adults with ASD into social, educational, and work settings, ultimately encouraging adults with ASD to use more social-based apps.

Overall, 6 parents and 1 specialist were interviewed in the poststudy interview. The interview focused on their experiences with the app, the challenges faced, and the recommended modifications. The open and axial coding phases of the grounded theory [39] were used to analyze the transcribed interview. The analysis highlighted the importance of the app in enabling communication between adults with ASD and other individuals. Most interviewees discussed the relevance of the app and its novelty. Five categories emerged from the analysis, which were app support, feature relevance, user interface design, overall feedback, and recommendations. Each category had subcategories and samples of excerpts from the transcribed data describing the experiences of the participants with ASD while using the app (Table 4).

**Table 4:** Categories and subcategories that emerged from the poststudy interview.

Category and subcategory			Description	Example quotes
**App support**			Technical and customized support to access the needed information without problems or errors	
	Learnability	Interactive app features, which aid participants with ASD^a^ to learn quickly		*...He was able to understand how to use it after I trained him...* [Parent #4]
	Efficiency (accessibility)	Accessing the features of the app (easy, medium, or hard)		*...He accepted the application very smoothly and enjoyed using it...* [Parent #4]
	Technical issues	Bugs or related technical issues		*...He tried to use recording, but it was a little hard for him to use it...* [Parent #5]
**Feature relevance**			Feedback of the participants on the app features	
	Text-to-speech	Feedback on the text-to-speech feature		…*it was an amazing experience for him to listen to the text, while this feature is not available in any other platform even WhatsApp...* [Parent #1]
	Speech-to-text	Feedback on the speech-to-text feature		…*Speech to text is a very good feature I would say especially for autistic case…* [Parent #6]
	Arabic issues	Participants talk about Arabic issues related to the app		…*The Arabic language was a little annoying since text-to-speech and speech-to-text transcriptions were robotic and need more advancements…* [Parent #2]
	TAWASOL symbols	Feedback on TAWASOL symbols		…*For him, both TAWASOL symbols and voice messaging are the main features that helped him…* [Parent #2]
**User interface design**			Visual appearance of the app, such as the arrangement of content, color schemes, icons, and font sizes	
	Color	Feedback on the color changing tool of the app		…*He didn’t change the application color nor the chat bubble since he doesn’t have any problem with colors, but other autistic individuals can use this feature to help them using the application more by changing the color with what is suitable for them…* [Parent #2]
	Design consistency	The participant likes texting and using the app like other messaging apps		…*He knows [refereeing to the participants with ASD] how to type and use it in other application, then he liked the chatting (Texting) part more…* [Parent #1]
**Overall feedback**			Feedback about the app in general regarding the innovative idea and the need of it	
	Positive feedback	Positive feedback on the app		…*Really good application for chatting like I'm very happy about the way it sends messages in different format text-to-speech, speech-to-text, and Tawasol symbols, so I was introducing it to him as a chatting app itself and he liked it* [Caregiver #1]
	Novelty	Feedback on the novelty of the app compared to existing messaging apps		…*I’m just saying because it's a very good with its unique features what I really found very interesting since I can’t find these features in other similar applications such as WhatsApp…* [Parent #1]
**Recommendations**			Suggestions of parents and specialists on how to improve the app	
	Serious games	Suggestions to enhance the app with game elements to teach specific skills and knowledge		…*I would like to suggest including games where autistic adults could learn the TAWASOL symbols and be able to easily construct sentences…* [Parent #3]
	Privacy protection	Discussing the next version based on a friend request feature in order to protect users from strangers		…*Caregivers should be able to accept friends, and this is because we need to protect them from unknown people…* [Parent #3]

^a^ASD: autism spectrum disorder.

## Discussion

### Summary

In this study, MAAN, a mobile messaging app, was developed and evaluated in 7 adults with ASD over 10 to 16 weeks. A pre-post study was also conducted with experts and parents on the interface and functionality design of the app. The app has additional features (TTS, STT, and CS) when compared with existing messaging apps. MAAN is designed to support adults with ASD when communicating with other individuals via text messaging in both Arabic and English. It offers the ability for adults with ASD to read or listen to text messages and then reply. The results supported the hypotheses and are encouraging for further work in the future.

### Comparison With Related Work

Very few messaging apps that support social and communication skills in individuals with ASD are discussed in the literature. TalkingBoogies [42], an app introduced in 2020 by a research team in Korea, aimed to actively assist caregivers when developing AAC-led communication with children having ASD. It comprises the following 2 iOS apps: TalkingBoogies-AAC for child caregiver communication and TalkingBoogies-Coach for caregiver collaboration. TalkingBoogies-AAC has several features, such as the use of Ewha [43], an AAC symbol system, and TTS. The team evaluated the developed app in 4 children with ASD and 3 teachers with at least 6 months of experience with AAC from a local special education school. The study concluded that such an app can prompt the learnability of children when constructing sentences. Prior to this study, De Leo et al [44] built and evaluated a Windows-based mobile app named “PixTalk.” The PixTalk system employs the PECS intervention and is made up of the following 2 modules: PixTalk smartphones, which enables children with ASD to search and select images in order to express their needs and feelings, and PixTalk website, which allows caregivers and teachers to add different images to a child’s smartphone. By conducting a case study involving 3 children with ASD and their teachers, this research asserted the importance of computer-assisted instructions as an intervention to motivate and engage learners with ASD. Proloquo2Go [45] and Proloquo4Text [46] are popular communication apps that use symbols and the TTS feature. Both apps are commercially available and widely used in school settings where English is the main instructional language. Nowadays, virtual communication platforms, such as social media networks and messaging apps, are becoming more integrated in most people’s lives [47]. However, from an accessibility viewpoint, the use of these platforms is neither practical nor accessible for the majority of individuals with ASD and especially for the nonverbal population. Some of the existing work involves AACrobat [48]. The authors designed this mobile app to help neuromuscular disease users communicate with others using their eyes. Although AACrobat uses the TTS feature, this apps is not helpful for individuals with ASD since most of them cannot fix their eyes in one direction to type from a keyboard or do not have the ability to generate a sentence. Currently, the most popular communication platform is WhatsApp [47], which is mainly used as a messaging app. Despite all the important updates and features that WhatsApp provides, it is still not accessible for individuals with ASD, as most of the nonverbal population struggles to read, write, or communicate using basic texting features. To the best of our knowledge, previous studies have attempted to support adults with ASD in constructing sentences for education and learning purposes, but no study has evaluated TTS, STT, and CS features, as well as social and family loneliness in adults with ASD. This paper presents the development and evaluation of the new messaging app MAAN, which means “together” in Arabic. MAAN employs TTS, STT, and CS to enhance the social and communication skills of adults with ASD. This is the first study to consider these features in a mobile messaging app designed to support communication among adults with ASD, both in English and Arabic. The results showed that MAAN can enhance social and communication skills, especially through distance messaging, and can ease communication with peers through its special features (TTS, STT, and CS). Hence, this study showed the effectiveness of these features in a messaging app.

### Principal Results

The 5 experts interviewed in the prestudy phase emphasized the importance of MAAN as a unique tool in supporting adults with ASD. During the interview, they discussed the app’s novelty and suggested several modifications, such as categorizing TAWASOL symbols and fixing the app’s design. Moreover, they emphasized the support of the app for the inclusion of adults with ASD into social, educational, and work settings, ultimately encouraging adults with ASD to use more social-based apps. On the other hand, the poststudy interview with parents suggested that the app provides 3 features directed to verbal and nonverbal adults with ASD, and the TTS feature was the most preferable feature for both ASD and non-ASD participants. Using MAAN helped the participants with ASD interact more with their parents, especially when they were not near them. Besides, the participants with ASD were attached to the app, where they voiced their needs by sending a message to their parents. Support for the Arabic language in all 3 features made this app very appealing to the participants, considering that there is a lack of ACC apps that support the Arabic language. Moreover, the interviewees highlighted the importance of MAAN in enhancing the social and communication skills of different users, such as individuals with dyslexia. From the participants’ engagements with the app and the SELSA-S questionnaire results, it can be deduced that more use of the app features was associated with higher SELSA-S questionnaire scores. Moreover, this was confirmed in the poststudy interviews where the interviewees highlighted the importance of the app features and their ability to increase the attention of adults with ASD, which can positively improve social loneliness. This development is noticeable with the positive engagement and enthusiasm that the participants with ASD exhibited when messaging their parents using the app features or when constructing a sentence by using TAWASOL symbols

### Limitations

Despite this study’s contributions, some limitations, including the sample size, operating system platform, and choice of features, were noted. Due to the COVID-19 pandemic, most adults with ASD were at home with their families and thus could not be easily reached. This led to a rather small sample. Another factor that contributed to the small sample was the absence of focused institutions for adults with ASD, since most of the current institutions serve children with ASD. A larger sample size could give more insights into the effects of the app and could increase the statistical power. Further, the message app was only available on the iOS platform. This availability limitation was due to the timeframe for developing the app. Lastly, other messaging app features, such as image messaging, video messaging, and voice and video calls, were excluded. The reason is to prompt adults with ASD to use AAC-based features rather than video or voice calls, which, in turn, can support their social and communication skills.

### Conclusion

The novelty of MAAN as a communication and social intervention app is its potential to support communication skills and social loneliness in adults with ASD. More importantly, it was possible to achieve this due to the inclusion of experts in the design and development of the app. Additionally, the poststudy evaluation by parents and specialists identified the uniqueness of the app, and how it could be enhanced and extended to other populations who also exhibit social and communication deficits, such as people with dyslexia. Future studies can consider a larger number of participants with ASD to replicate the findings and can extend this study to other clinical populations with social and communication deficits.

## Abbreviations

AAC: augmentative and alternative communication
ABA: applied behavioral analysis
ASD: autism spectrum disorder
CS: communication symbols
PECS: picture exchange communication system
SELSA-S: short version of the Social and Emotional Loneliness Scale for Adults
STT: speech-to-text
TTS: text-to-speech
uMARS: Mobile Application Rating Scale: user version
